# Supplementary material for: Racial and ethnic variation in COVID-19 care, treatment, and outcomes: A retrospective cohort study from the MiCOVID-19 registry
Source: PLoS One. 2022 Nov 1;17(11):e0276806. doi: 10.1371/journal.pone.0276806 (PMC9624408; doi:10.1371/journal.pone.0276806)
Supplement: S1 Table — (DOCX) [file pone.0276806.s001.docx]

Supplemental Table 1. In-hospital treatment for COVID-19 with remdesivir and dexamethasone by race and ethnicity, stratified by hospital size, teaching versus non-teaching hospital, and the surrounding population density of the hospital.

|  | Black | White | Asian | Latino | Other/ Unknown | p-value |
| --- | --- | --- | --- | --- | --- | --- |
| No. (%) | 1129 (43) | 1157 (44) | 54 (2) | 102 (4) | 197 (7) |  |
| **Hospital bed size < 250 beds** | | | | | | |
| Remdesivir In-Hospital Treatment | 0 (0) | 46 (12) | 0 (0) | 15 (24) | 5 (9) | < 0.001 |
| Dexamethasone In-Hospital Treatment | 15 (8) | 75 (20) | 0 (0) | 12 (19) | 11 (19) | < 0.001 |
| **Hospital bed size ≥ 250 beds** | | | | | | |
| Remdesivir In-Hospital Treatment | 25 (2) | 90 (11) | 4 (11) | 6 (15) | 14 (10) | < 0.001 |
| Dexamethasone In-Hospital Treatment | 30 (3) | 91 (12) | 3 (2) | 9 (23) | 11 (8) | < 0.001 |
| **Teaching Hospital** | | | | | | |
| Remdesivir In-Hospital Treatment | 25 (2) | 113 (11) | 4 (9) | 7 (12) | 16 (9) | < 0.001 |
| Dexamethasone In-Hospital Treatment | 40 (4) | 136 (13) | 3 (6) | 13 (22) | 20 (11) | < 0.001 |
| **Non-Teaching Hospital** | | | | | | |
| Remdesivir In-Hospital Treatment | 0 (0) | 23 (19) | 0 (0) | 14 (33) | 3 (25) | < 0.001 |
| Dexamethasone In-Hospital Treatment | 5 (9) | 30 (24) | 0 (0) | 8 (19) | 2 (17) | 0.11 |
| **Hospital Location** | | | | | | |
| Metropolitan | 1128 (100) | 1096 (95) | 54 (100) | 102 (100) | 193 (98) |  |
| Micropolitan | 1 (0) | 61 (5) | 0 (0) | 0 (0) | 4 (2) |  |
